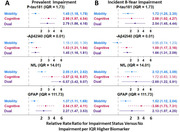# Associations of Alzheimer’s and Neurodegeneration Blood Biomarkers with Prevalent and Incident Mobility and Cognitive Impairment

**DOI:** 10.1002/alz.086174

**Published:** 2025-01-09

**Authors:** B Gwen Windham, Chad T Blackshear, Farwa Ali, David S. Knopman, Keenan A. Walker, James Russell Pike, Yifei Lu, Thomas H. Mosley, Priya Palta, Michael E. Griswold

**Affiliations:** ^1^ University of Mississippi Medical Center, The MIND Center, Jackson, MS USA; ^2^ Mayo Clinic, Rochester, MN USA; ^3^ Laboratory of Behavioral Neuroscience, National Institute on Aging, Intramural Research Program, Baltimore, MD USA; ^4^ New York University, New York, NY USA; ^5^ University of North Carolina Chapel Hill, Chapel Hill, NC USA; ^6^ University of Mississippi Medical Center, Jackson, MS USA

## Abstract

**Background:**

Older adults with dual cognitive and mobility impairments have higher financial costs and poorer quality of life than adults with either impairment alone. Blood biomarkers of Alzheimer Disease (AD) pathology (Aβ42, Aβ40 and p‐tau181) and neurodegeneration (neurofilament light (NFL) and glial fibrillary acidic protein (GFAP)) may identify individuals at risk for both mobility and cognitive impairment and provide novel insights into mechanistic underpinnings.

**Method:**

Blood biomarkers (SiMoA Quanterix N4PE, p‐tau181 single‐plex) were available in a subsample of 1751 ARIC study participants at Visit 5 (V5, 2011‐13, mean age 76.2 years (5.3), 41% men, 28% Black); Aβ42 was reversed and adjusted for Aβ40 (‐Aβ42|40) for consistent directions of associations (higher=worse). Usual gait speed and cognitive status were assessed over 8 years at V5, V6 (2016‐17), and V7 (2017‐18). Participants were categorized by V5 impairment: mobility‐only (gait speed <0.8m/s, n=430), cognitive‐only (expert‐adjudicated dementia, n=38), dual (n=54), or neither (referent, n=1229). Multinomial regression models examined relative risk ratios (RRR) of V5 log‐transformed biomarkers with prevalent impairments and, among those with neither impairment at V5, incident impairments through V7. All models adjusted for V5 age, sex, self‐reported race (Black or White), renal function, and body mass index.

**Result:**

Higher levels of all biomarkers were associated with greater risk of prevalent impairment in mobility, cognitive, or dual domains (Figure A). Over 8‐years of follow‐up, incident impairments included 471 participants with only mobility impairments, 39 with only cognitive impairments, 55 with dual impairments, and 168 died without developing any impairment. All biomarkers were associated with incident dual impairment per IQR higher biomarker: p‐tau181 (RRR=2.54; 95% CI: 1.45, 4.44), ‐Aβ42|40 (RRR=1.66; 1.31, 2.09), NfL (RRR=2.69; 1.22, 5.91) and GFAP (RRR=2.13; 1.07, 4.25). P‐tau181, ‐Aβ42|40, and GFAP were also associated with incident cognitive‐only impairment, while P‐tau181, NFL and GFAP were associated with incident mobility‐only impairment. (Figure B).

**Conclusion:**

Blood biomarkers of brain health may be useful tools for earlier identification of those at risk for cognitive, mobility, or dual declines, facilitating earlier targeted interventions. Potential differences in AD‐pathology versus neurodegeneration blood biomarker relationships with outcomes of mobility and cognition may help to identify more targeted interventions for both.